# A randomized trial comparing the Tennant Biomodulator to transcutaneous electrical nerve stimulation and traditional Chinese acupuncture for the treatment of chronic pain in military service members

**DOI:** 10.1186/s40779-019-0227-4

**Published:** 2019-12-02

**Authors:** Kimberly S. Peacock, Erika Stoerkel, Salvatore Libretto, Weimin Zhang, Alice Inman, Michael Schlicher, John D. Cowsar, David Eddie, Joan Walter

**Affiliations:** 1 University of Texas Health San Antonio, 7703 Floyd Curl Drive, San Antonio, TX 78229 USA; 2 TLI Foundation, 1750 Tysons Boulevard, McLean, Virginia, 22102 USA; 3 Henry M. Jackson Foundation, 6720A Rockledge Drive, Bethesda, MD 20817 USA; 4 BD Life Sciences, 1 Becton Drive, Franklin Lakes, NJ 07417 USA; 5 Brooke Army Medical Center, 3551 Roger Brooke Drive, Fort Sam Houston, TX 78234 USA; 6 Madigan Army Medical Center, 9040A Jackson Ave, Joint Base Lewis-McChord, WA 98431 USA; 7 MedPost Urgent Care Center, 513 Cibolo Valley Drive, Cibolo, TX 78108 USA; 8 Massachusetts General Hospital, Harvard Medical School, 151 Merrimac St. 6th Floor, Boston, MA 02114 USA; 9 H&S Ventures, Samueli Integrative Health Program, 1800 Diagonal Road, Suite 617, Alexandria, VA 22314 USA

**Keywords:** Tennant biomodulator, Acupuncture, Transcutaneous electrical nerve stimulation, Chronic pain, Military service members

## Abstract

**Background:**

The present investigation tested the efficacy of the Tennant Biomodulator, a novel pain management intervention that uses biofeedback-modulated electrical stimulation, to reduce chronic pain and its psychosocial sequelae in a sample of current and former military service members. The Tennant Biomodulator used on its most basic setting was compared to two commonly used, non-pharmacological pain treatments—traditional Chinese acupuncture and transcutaneous electrical nerve stimulation (TENS)—in a comparative efficacy, randomized, open-label trial.

**Methods:**

Participants included 100 active duty and retired service men and women with chronic pain undergoing treatment at the Brooke Army Medical Center in Texas, USA, randomly assigned to receive six, weekly sessions of either Tennant Biomodulator treatment, traditional Chinese acupuncture, or TENS, in addition to usual care. Recruitment was conducted between May 2010 to September 2013. Outcome measures were collected at intake, before and after each treatment session, and at a 1-month follow-up. Intent-to-treat analyses were used throughout, with mixed models used to investigate main effects of group, time, and group × time interactions with consideration given to quadratic effects. Outcomes measured included ratings of chronic pain, pain-related functional disability, and symptoms of post-traumatic stress disorder (PTSD) and depression.

**Results:**

On average, regardless of their treatment group, participants exhibited a 16% reduction in pain measured by the Brooke Army Medical Center’s Clinic Pain Log [*F*(1, 335) = 55.7, *P* <  0.0001] and an 11% reduction in pain-related disability measured by the Million Visual Analog Scale [MVAS: *F*(1, 84) = 28.3, *P* <  0.0001] from baseline to the end of treatment, but no one treatment performed better than the other, and the reductions in pain and pain-related disability were largely lost by 1-month follow-up. Symptoms of PTSD and depression did not change significantly as a function of time or group.

**Conclusions:**

Findings build on previous work suggesting that traditional Chinese acupuncture and TENS can reduce pain and its functional sequelae without risks associated with pharmacological pain management. The Tennant Biomodulator used on its most basic setting performs as well as these other interventions. Based on the present findings, large, randomized controlled trials on the Tennant Biomodulator are indicated. Future work should test this device using its full range of settings for pain-related psychological health.

**Trial registration:**

Clincialtrials.gov (NCT01752010); registered December 14, 2012.

## Background

An estimated 126 million Americans suffer from chronic pain, leading to prodigious individual burden and costing the United States economy $635 billion dollars a year in loss of productivity and healthcare expenses [1, 2]. Surgery and/or pharmacotherapy can be effective for acute pain management, however, surgery typically involves risk for complications, and medications often have marked side-effect profiles. Furthermore, opioid-based pain medications also involve a risk for the development of opioid use disorder. In light of these limitations, as well as the current opioid crisis largely attributed to the over-prescription of opioid medications for pain management [3] and the enormous personal and public health burden of chronic pain, there is a pressing need for non-pharmacological, accessible, and scalable pain management interventions.

Certain subgroups of individuals are disproportionally affected by chronic pain, including military service men and women, and military veterans [4–6]. The importance of pain management for injured service members was highlighted by the Veterans Pain Act of 2007 [7], in which the United States Congress identified pain as a leading cause of short-term and long-term disability among veterans. Despite ongoing efforts to address this major problem, military personnel and veterans remain at elevated risk. For instance, larger amounts of prescribed opioids have been shown to increase risk of self-inflicted drug overdose deaths among military veterans with chronic pain [8]. Veterans are also thought to be disproportionately affected by substance use disorders arising from pain medication prescriptions and attempts to self-manage pain with alcohol and other drugs [9].

Chronic pain has also been shown to exacerbate symptoms of post-traumatic stress disorder (PTSD) [10–12], making military service members and veterans—who are more likely than the non-military population to have PTSD [13]—particularly vulnerable to the deleterious effects of chronic pain. Conversely, there is evidence that reducing chronic pain can help mitigate PTSD severity [10], reinforcing the case for aggressive yet safe pain management for military personnel and veterans.

Several non-pharmacological pain interventions have demonstrated efficacy in ameliorating chronic pain. For example, traditional Chinese acupuncture has been used extensively in pain management [14], with generally positive findings [14, 15]. While acupuncture has few contraindications, has a low side-effect profile, and is generally deemed safe, it lacks accessibility and scalability because it requires regular and ongoing treatment by medical acupuncturists.

In contrast, transcutaneous electrical nerve stimulation (TENS) devices offer better accessibility and scalability since they are relatively inexpensive, and they can be self-administered in the home. Conventional TENS units work by delivering localized, transdermal electrical stimulation between 1 and 80 mA (mA), which is thought to reduce pain by stimulating peripheral afferent A-β fibers, thus inhibiting A-delta and C-fiber-mediated nociception in the dorsal horn [16], while also stimulating endorphin production [17, 18]. TENS treatment for chronic pain, however, has produced mixed results in clinical trials [19], and taken together, the results in the literature are inconclusive about TENS’ effectiveness for managing chronic pain [20]. Additionally, repeated TENS administration may result in analgesic tolerance [21].

In light of the pressing need for effective, scalable, non-pharmacological pain interventions, particularly for military service members and veterans, the present study tested a novel pain-management treatment against traditional Chinese acupuncture and acupuncture-like TENS.

The hand-held Tennant Biomodulator® (Senergy Medical Group, Irving, Texas, USA) tested in this study is an FDA-cleared Class II device, which is increasingly being used for the management of chronic pain. This class of devices emits a pulsed, damped, biphasic, sinusoidal, transdermal electrical current between 20 and 500 microamperes (μA). Because of the very low current involved, administration of this treatment does not produce a significant amount of heat or interfere with nerve or muscle function.

The Tennant Biomodulator is a precision medical instrument requiring a doctor’s prescription. It has the capacity to operate on a range of settings, some of which are optimally administered by treatment providers. On its most basic setting, however, treatment with the device can be self-administered by patients at home. Because the Tennant Biomodulator is non-invasive and causes no pain, it is a safe pain management tool. Pilot testing of the Tennant Biomodulator has yielded largely positive findings [22–24], but the results of tests on other microcurrent stimulation devices have been mixed (e.g., [25–28]).

The present treatment superiority study sought to compare the efficacy of the Tennant Biomodulator used on its most basic setting against traditional Chinese acupuncture, and acupuncture-like TENS for the reduction of chronic pain and associated functional disability. Additionally, because physical pain is thought to exacerbate symptoms of PTSD [29] and depression [30], secondary objectives were to assess whether the Tennant Biomodulator improves PTSD and depression symptoms. It was hypothesized that the Tennant Biomodulator would produce larger reductions in pain and related functional disability among treatment-seeking military service members compared to traditional Chinese acupuncture or treatment with a standard TENS unit set to deliver acupuncture-like TENS over a 6-week treatment period. Evaluation of the effects of Tennant Biomodulator treatment on secondary outcomes, including PTSD symptoms and depression, were treated as exploratory. Thus, no a priori hypotheses were proposed for this aspect of the study, and Biomodulator device settings that were designed to optimize mental health outcomes were not used.

## Methods

Regulatory approvals for this study were obtained from the United States Army Department of Clinical Investigation, the Brooke Army Medical Center Institutional Review Board, and the Human Research Protection Office (HRPO) at the United States Army Medical Research and Materiel Command (IRB approval number C.2009.098). The protocol was registered prior to participant enrolment on clincialtrials.gov (ID: NCT01752010). All study staff completed relevant Collaborative Institutional Training Initiative (CITI) ethics training prior to study initiation and maintained certification throughout the trial period.

### Participants

Participants were military service members and military veterans who were seeking treatment for chronic pain conditions at the Brooke Army Medical Center’s Pain Clinic. Recruitment was conducted between May 2010 to September 2013. Inclusion criteria included being an injured military service member or veteran between the ages of 18 and 60 and having at least 3 months of current, chronic pain. Exclusion criteria included being pregnant or considering pregnancy within the study period, having a history of epilepsy, having a pacemaker or other implanted medical device, having a history of cardiac arrhythmias, having underwent surgical intervention during the past month for the treatment of lower back pain or its underlying etiology, having a documented history of prescription medication misuse, having misused illicit drugs within the last 6 months, and having participated in a clinical trial for an investigational drug within 30 days prior to screening. All study treatments were provided free of cost; participants were not compensated for their participation in the study. Based on the available research resources, a sample size of *n* = 30 per treatment arm was selected, with a total target sample size of *N* = 100 to offset drop-out.

### Measures

#### Clinic pain log

The Clinic Pain Log is a standard form utilized in the Brooke Army Medical Center’s pain clinic. Patients self-rated their level of pain before each treatment session and then again 30 min after each treatment session on a scale from 0 to 10, where 0 was defined as “no pain,” and 10 was defined as “severe pain.”

#### Million visual analog scale (MVAS)

The MVAS [31] is a 15-item visual analog measure that evaluates the effects of chronic pain on functional ability. This instrument focuses on pain-related function rather than pain severity and generates a total functional disability score ranging from 0 to 150 with the following heuristically defined categories: no reported disability (0), mild disability (1–40), moderate disability (41–70), severe disability (71–100), very severe disability (101–130), and extreme disability (131–150) [32].

#### PTSD checklist - military (PCL-M)

The PCL-M [33] is the Military Version of the PTSD Checklist designed to assess PTSD symptoms in military personnel and veterans. It contains 17 PTSD-related problems or complaints that are rated on a 5-point scale ranging from “not at all” to “extremely” in terms of how much the problems or complaints have affected the individual over the past month. The composite scores can range from 17 to 85. The PCL-M has excellent internal consistency (α = 0.93) and test-retest reliability (*r* = 0.96) [34].

#### The Center for Epidemiological Studies - depression scale (CES-D)

The CES-D [35] measures depression symptom severity using a 20-item scale that yields total scores ranging from 0 to 60. Scores above 16 are indicative of major depression. The CES-D measure has excellent internal consistency (α = 0.90) and equivocal test-retest reliability (*r* = 0.54) [35].

### Procedure

After informed consent was obtained, participants were randomized using allocation concealment via a computer-generated integer generator to receive either treatment with the Tennant Biomodulator, traditional Chinese acupuncture, or TENS. Given the open label nature of the study, participants and providers were not blinded to treatment condition.

At baseline, all participants completed pain (clinic pain log), functional disability (MVAS), PTSD (PCL-M), and depression (CES-D) assessments, as well as demographic questionnaires, and provided detailed medication histories. The MVAS, PCL-M, and CES-D were readministered at the end of 6 weeks of active treatment and again at the one-month follow-up.

Participants who reported baseline scores > 44 on the PCL-M PTSD assessment or > 16 on the CES-D depression assessment were referred to the Behavioral Medicine Department at the study site for a more complete evaluation and were allowed to participate in the study. Pain and pain-related functionality were assessed before and after each clinical session using the Clinic Pain Scale and the MVAS.

All participants attended one session weekly over the 6-week treatment period with a licensed medical doctor who was specialized in pain management and trained in the use of the three treatment modalities. Sessions lasted for 20 min for Biomodulator and TENS treatments, and for 30 min for acupuncture treatments. The sessions included a brief check-in with the treatment provider, pain treatment administered by the provider, and for those in the Biomodulator and TENS groups, the provision of instructions for home use of their study instrument for the remainder of the six weeks in the study period.

### Treatments

All study patients received usual care, including physical therapy and nonopioid pain medication, which was individualized based on patient presentations and needs. Study treatments were provided as adjunctive care, based on random group assignments. All study patients were seen by the same pain doctor for the duration of the study, and the doctor was blinded to the subject reports of any pain improvement.

#### Tennant Biomodulator

Patients randomly assigned to receive treatment with the Tennant Biomodulator were taught how to use the device for basic pain relief.  In each clinic session four electrodes, using two channels, were placed on the proximal, distal, medial and lateral borders of the region of pain, with stimulation applied for 20 min. Weekly clinical sessions included reassessments by the treating provider, a Biomodulator treatment, and reinforcement of the home use instructions. The participants took the Biomodulator home and were instructed to use the machine twice a day over the course of the six-week treatment period. Participants were taught to place the device (with or without the adhesive electrodes) over the area of pain and then increase the electrical current output until they felt a slight tingle. Participants choosing to use the device without the adhesive electrodes (i.e., using the electrodes embedded within the device) were instructed to lightly press the device onto the site of pain and rotate it in a counter-clockwise direction. Participants were told to place the device on the contralateral side after one minute and repeat these steps, for a total treatment administration time of 15 min.

#### Traditional Chinese acupuncture

Participants in the traditional Chinese acupuncture group provided the study doctor with a clinical history of their pain problems before receiving a brief physical exam of the symptomatic area, which included a range of motion exam and a palpatory exam of the affected site; any areas of tenderness were specifically noted (i.e., Ah Shi or trigger points) [36]. Traditional Chinese medicine diagnostic methods, such as examining the patient’s tongue or pulse for Ying/Yang abnormalities, were not used. During the weekly treatment sessions, needles were placed based on the traditional Chinese acupuncture points described by Worsley [37] and the Ah-Shi points. No auricular points were involved in this study. Standard Chinese sterile steel acupuncture needles were used with two-channel electrical stimulation and intermittent manual stimulation of the needles.

While the location of participants’ pain determined the specific set of acupuncture points used, the same points were used on all participants experiencing pain in the same area. For back pain without radicular pain, standard traditional Chinese lumbosacral points were used. Participants with radiculating lower back pain spreading down the leg were also treated via traditional radicular pain points on the leg. Approximately 5 s of manual stimulation (tonification) was used for all needles immediately following insertion and then again at five-minute intervals for the needles not connected to electrodes. For the needles connected to electrodes, 2 Hz electrical stimulation was used. All participants received a combination of manual and electrical needle stimulation. At the end of each session, the needles were again manually stimulated for approximately 5 s each before they were removed. All patients who were randomly assigned to the acupuncture treatment arm were treated by the same medical acupuncturist, and the needles were left in place for 20–30 min.

#### TENS

Patients randomly assigned to receive acupuncture-like TENS were treated by a physician at weekly visits using two channels with a total of four electrodes placed on the proximal, distal, medial, and lateral borders of the region of pain in a standard manner. Based on the Brooke Army Medical Center Pain Clinic standard operating procedure for TENS units, the frequency setting used was 2 Hz with a pulse width of 175–200 microseconds. The intensity was set to the maximum tolerated, with a treatment duration of 20 min. Participants were provided an Empi™ TENS (Empi, St. Paul, MN, USA) device powered by a 9-Volt battery and were instructed to use the machine twice a day over the course of the 6-week treatment period.

### Analyses

Intent-to-treat analyses were conducted. The analyses therefore included all participants, including those who attended only one session after signing the informed consent form and were randomized to a treatment group (*n* = 10). However, those who failed to attend at least one session after they provided informed consent were not included in the analyses. These intent-to-treat data provided a more complete picture of the study outcomes regardless of whether the participant completed the 6 weeks of the study treatments. Mixed models [38] were used to investigate main effects of time on pain (clinic pain log), and pain-related impairment of functioning (MVAS). Main effects of group, and group × time interactions were also tested with consideration of quadratic effects. The covariance structure of the model was chosen based on Akaike’s Information Criterion (AIC). Mixed models are preferable to traditional general linear models because they consider all available data and do not rely on data imputation procedures or listwise deletion for missing data. The spatial exponential covariance structure was used to assess differences in time between repeated measurements. We added a measurement error component to the serial correlation component and eliminated the second derivatives when calculating the covariance matrix adjustment. When significant main and interaction effects were detected, least square mean post hoc tests were performed, with Tukey-Kramer corrections for test-wise alpha level inflation.

Within-session change in pain was calculated by subtracting participants’ mean pain ratings recorded at the end of each clinical session from their pain ratings recorded before each clinical session.

Changes in PTSD (PCL-M) and depression symptoms (CES-D) were explored using linear mixed regression models with a compound symmetry covariance structure. Main effects of time, group, and group × time interactions were tested.

All analyses were conducted using SAS (version 9.2, SAS Institute, Cary, NC, USA) [39] and *P* <  0.05 was considered statistically significant.

## Results

The characteristics of the participants are described in Table 1. In brief, the average age of the participants was 45.8 years of age (*SD* = 10.8), 22 (22%) participants were female, 24 (24%) participants were African American, 43 (43%) were European American, and 28 (28%) idenfitied as other. On average, participants reported having experienced chronic pain for 7.9 years at study entry (*SD* = 8.3), and the number of years of pain was positively associated with age (*r* = 0.3, *P* <  0.01). The treatment groups were not significantly different in terms of the demographic characteristics with the exception of the marital/relationship status; most participants in the TENS group were married or in a relationship (*n* = 27; 90%), but only 22 (61%) participants in the acupuncture group were married or in a relationship (*P* <  0.01). Forty participants (40%) were active duty soldiers, two (2%) were army reservists, two (2%) were in the National Guard, and 48 (48%) were retired.

**Table 1:** Participant characteristics (*n* = 100)

	Group			*F* / *χ*^*2*^
	Biomodulator (*n* = 34)	Acupuncture (*n* = 36)	TENS (*n* = 30)	
Age (year, mean ± SD)	49.1 ± 10.8	45.8 ± 10.6	42.2 ± 10.3	4.5
Females [*n* (%)]	7 (20.6)	9 (25.0)	6 (20.0)	0.3
Race^**^ [*n* (%)]				2.5
African American	7 (20.6)	11 (30.6)	6 (20.0)	–
European American	15 (44.1)	12 (33.3)	16 (53.3)	–
Other	8 (23.5)	12 (33.3)	8 (26.7)	–
Married or in a relationship [*n* (%)]	23 (67.6)	22 (61.1)	27 (90.0)	6.6^*^
Bachelor’s degree or higher [*n* (%)]	21 (61.7)	19 (52.8)	18 (60.0)	0.3
Active Duty [*n* (%)]	10 (29.4)	15 (41.7)	15 (50.0)	0.2
Years of pain (mean ± SD)	8.6 ± 6.5	8.6 ± 10.5	6.3 ± 7.0	4.3
Taking medication [*n* (%)]	25 (73.5)	22 (61.1)	19 (63.3)	0.4

-. No data; *. *P* <  0.05; **. Race data missing for 5 participants; TENS. Transcutaneous electrical nerve stimulation

Participants were primarily seeking treatment for musculoskeletal pain, with the following locations were considered the primary body areas requiring attention: back, 50 (50%); a combination of musculoskeletal areas, 23 (23%); neck, 4 (4%); shoulder, 4 (4%); hand/wrist, 2 (2%); hip, 2 (2%); knee, 2 (2%); legs, 3 (3%); ankle, 1 (1%); and elbow, 1 (1%). Migraine was reported as the primary cause of pain in 1 (1%) participant. Seven (7%) of the participants did not report a primary area of pain. Most participants indicated that their pain was a result of injuries sustained while on active duty in the military, either during the course of day-to-day operations (e.g., carrying heavy weaponry, training accidents, falls) or in combat (e.g., improvised explosive device blasts).

Of the 100 military service members who were recruited to participate in the study and signed the informed consent form, 34 were randomly assigned to receive Biomodulator treatment, 36 were randomly assigned to receive traditional Chinese acupuncture, and 30 were randomly assigned to receive TENS. Five participants signed the informed consent form but never attended treatment; four of these participants were assigned to the Biomodulator treatment arm, and one was assigned to the acupuncture arm. Ten participants completed only one session, 10 completed only two sessions, and 10 completed only three sessions. The total study attrition rate, with attrition defined as ≥4 clinical sessions missed, was 27% (*n* = 27). The attrition rates by treatment arm were as follows: Biomodulator, 32% (*n* = 11); acupuncture, 36% (*n* = 13); and TENS, 10% (*n* = 3). This difference was statistically significant, *χ*^*2*^ (2, *n* = 100) = 10.3, *P* = 0.006. The reasons for study drop-out were not formally assessed; however, it was understood that most of the active duty soldiers dropped out because of a deployment order, and no adverse events were reported in relation to the administration of the Biomodulator, traditional Chinese acupuncture, or TENS device in the study.

The severity of the participants’ self-reported pain, PTSD and depression symptoms are reported in Table 2.

**Table 2:** Participants’ self-reported pain and pain related functional disability (pain log & MVAS), PTSD (PCL-M) and depression symptom severity (CES-D) by group and visit number [*n*, mean ± SD]

Item	Visit 1	Visit 2	Visit 3	Visit 4	Visit 5	Visit 6	Follow-up visit
Pain log (pre-session)							
Biomodulator (*n* = 34)	25, 4.7 ± 1.9	17, 3.8 ± 2.5	18, 3.8 ± 2.1	17, 4.2 ± 2.0	13, 3.5 ± 1.7	13, 3.1 ± 1.4	13, 3.7 ± 2.3
Acupuncture (*n* = 36)	32, 4.6 ± 1.9	20, 3.9 ± 1.8	21, 3.5 ± 2.2	19, 2.9 ± 2.2	18, 3.8 ± 2.5	18, 3.2 ± 1.5	11, 3.4 ± 3.0
TENS (*n* = 30)	26, 4.5 ± 1.8	23, 3.5 ± 1.5	22, 3.5 ± 1.8	21, 3.4 ± 1.8	21, 2.7 ± 1.8	22, 2.7 ± 1.4	9, 3.3 ± 1.4
Pain log difference [(pre-session) – (post-session)]							
Biomodulator (*n* = 34)	25, −1.8 ± 1.7	17, − 0.9 ± 2.0	16, − 1.4 ± 1.6	17, −1.9 ± 1.6	12, − 1.3 ± 1.4	13, −1.6 ± 0.9	–
Acupuncture (*n* = 36)	32, − 1.8 ± 1.7	20, − 1.6 ± 1.5	21, −1.3 ± 1.6	18, − 0.9 ± 1.2	18, −1.6 ± 1.8	17, − 1.2 ± 0.8	–
TENS (*n* = 30)	24, − 1.8 ± 2.3	22, −1.4 ± 1.1	22, − 1.0 ± 1.4	21, −1.3 ± 1.1	21, − 0.8 ± 0.8	21, − 1.0 ± 0.7	–
MVAS							
Biomodulator (*n* = 34)	30, 81.2 ± 20.5	20, 68.9 ± 25.0	16, 64.1 ± 24.3	15, 68.4 ± 24.5	13, 64.8 ± 27.5	15, 59.1 ± 29.9	21, 59.1 ± 29.8
Acupuncture (*n* = 36)	35, 72.2 ± 25.5	22, 54.5 ± 25.4	19, 54.7 ± 25.7	20, 51.3 ± 30.9	17, 56.2 ± 33.6	16, 54.5 ± 28.9	19, 54.1 ± 33.4
TENS (*n* = 30)	29, 67.14 ± 18.3	19, 68.9 ± 25.1	25, 63.4 ± 26.9	19, 65.6 ± 27.4	17, 58.6 ± 25.0	22, 57.1 ± 25.2	25, 50.0 ± 22.5
PCL-M							
Biomodulator (*n* = 34)	25, 35.2 ± 15.2	–	–	–	–	15, 38.6 ± 17.6	13, 37.2 ± 15.9
Acupuncture (*n* = 36)	33, 31.7 ± 18.1	–	–	–	–	14, 30.1 ± 17.6	15, 37.8 ± 21.9
TENS (*n* = 30)	26, 27.5 ± 10.5		–	–	–	16, 25.9 ± 13.1	18, 25.2 ± 11.8
CES-D							
Biomodulator (*n* = 34)	22, 18.3 ± 11.3	–	–	–	–	15, 23.5 ± 14.7	14, 20.8 ± 14.6
Acupuncture (*n* = 36)	33, 12.1 ± 12.7	–	–	–	–	12, 16.7 ± 16.0	18, 18.4 ± 14.9
TENS (*n* = 30)	26, 10.7 ± 10.3	–	–	–	–	15, 11.2 ± 12.8	16, 12.6 ± 10.8

-. No data; CES-D. The Center for Epidemiological Studies - Depression Scale; MVAS. Million Visual Analog Scale; PCL-M. PTSD Checklist - Military; PTSD. Post-traumatic stress disorder; TENS. Transcutaneous electrical nerve stimulation

The results from mixed models are presented in Table 3. Clinical pain log scores capturing pre-session pain showed a significant main effect of time, as the mean pain scores decreased over time. There was also a significant time quadratic effect, indicating that pain log scales decreased during treatment but then increased again by the follow-up. The group × time interaction was not statistically significant (*P* > 0.05).

**Table 3:** Results from the mixed models showing main effects of group and time, the time-quadratic effect, and the group × time interaction effect on outcome measures

Item	*df*	*F*	*P*
Clinic Pain Log (pre-session)			
Group	2, 107	0.3	NS
Time	1, 84	28.3	< 0.0001^*^
Time (quadratic)	1, 104	16.8	< 0.0001^*^
Group × Time	2, 61	0.5	NS
Clinic Pain Log (change within session)			
Group	2, 156	0.0	NS
Time	1, 195	5.4	0.02^*^
Time (quadratic)	1, 258	2.4	NS
Group × Time	2, 138	1.2	NS
MVAS			
Group	2, 98	4.0	0.02^*^
Time	1, 335	55.7	< 0.0001^*^
Time (quadratic)	1, 331	28.2	< 0.0001^*^
Group × Time	2, 335	4.7	0.01^*^
PCL-M			
Group	2, 91	1.7	NS
Time	2, 89	0.1	NS
Group × Time	4, 89	0.7	NS
CES-D			
Group	2, 97	2.8	NS
Time	2, 89	2.3	NS
Group × Time	4, 89	0.3	NS

*. *P* < 0.05; CES-D. The Center for Epidemiological Studies - Depression Scale; *df*. degrees of freedom; MVAS. Million Visual Analog Scale; NS. not significant; PTSD. Post-traumatic stress disorder; PCL-M. PTSD Checklist - Military

For pre- to post-session change in pain scores, mixed models showed a significant main effect of time, with mean pain scores decreasing over time. The time quadratic and group × time interaction effects were not statistically significant (*P* > 0.05).

MVAS scores showed a significant main effect of time, with mean MVAS scores decreasing over time. A significant time quadratic effect was also observed, indicating that MVAS scores decreased during treatment but then plateaued or increased again by the time of the follow-up visit. There was also a main effect of group; however, post hoc tests did not reveal significant between-group differences after Tukey-Kramer correction. A significant group × time interaction effect was also observed; however, post hoc tests did not show any significant differences between groups within each visit. In absolute terms, regardless of treatment group, the six participants who reported having primary lower back pain at baseline experienced an average reduction in MVAS scores of 13.6 (*SD* = 21.3) points at follow-up, while on average participants who reported having primary pain in other musculoskeletal areas (e.g., combination of musculoskeletal areas, neck, shoulder, hand/wrist, hip, knee, legs, ankle, elbow, migraine) experienced 14.5 (*SD* = 18.3) point reductions in MVAS scores.

We performed a post hoc power analysis using the SAS GLMPOWER procedure [40] to calculate the statistical power required to detect between-group differences in the MVAS scores throughout the course of the study. By specifying a base correlation of 0.8, a decay rate of 0.9, a standard deviation of 26, a given sample size of *n* = 60 and seven repeated measures, we achieved 20% power to detect a main effect of group, and 94% power to detect a group × time interaction effect.

According to the mixed model tests, there were no significant effects of the treatment on PTSD symptoms (PCL-M) or depression symptoms (CES-D) (*P* > 0.05).

## Discussion

In light of the enormous personal and public health burden of chronic pain and the current opioid crisis in the United States, the present investigation sought to test the efficacy of the Tennant Biomodulator, an accessible and scalable pain management intervention with a minimal side-effect profile and few contraindications. While the Biomodulator can be operated with a range of settings, we tested it here on its most basic setting for in-home use against traditional Chinese acupuncture and acupuncture-like TENS for the reduction of chronic pain and associated functional disability.

All three interventions produced significant reductions in chronic pain over 6 weeks of treatment, as evinced by a main effect of time on the clinic pain scores. A time quadratic effect was also observed, indicating that pain log scores decreased over the course of treatment but then increased again by 1-month follow-up, though notably, scores did not increase to baseline levels. There was, however, no group main effect or group × time interaction effect for this measure, indicating that no one treatment outperformed the others in terms of chronic pain management. Similarly, a main effect of time was also observed in the pre- to post-session change in the pain score (i.e., change in pain from the beginning to the end of individual clinical sessions), showing the mean pain change scores decreased over time. The diminishing reduction in the change in pain scores within sessions over the treatment period is most likely explained by decreasing pre-session pain scores (i.e., patients were starting sessions with less pain, so they were likely to report smaller within-session pain reductions), but it may also reflect diminishing treatment effects over the six treatment sessions. No main effect of group, or group × time interaction was observed for the pre- to post-session pain score change, indicating that no one treatment outperformed the others in terms of the within-session pain score reductions.

Reductions in pain-related functional disability were also observed, as indicated by a significant main effect of time on the MVAS scores, such that mean MVAS scores decreased over time for participants in all three groups. A significant time quadratic effect was also observed, showing that the MVAS scores decreased during treatment but then plateaued or increased again by the time of the follow-up visit, which occurred one month later. A main effect of group was also observed; however, this effect was not significant after Tukey-Kramer correction for alpha inflation, suggesting that no one treatment outperformed the others in terms of improving pain-related functional ability. A significant group × time interaction effect was observed, but the post hoc tests did not show any significant differences between groups within each visit.

No significant changes were observed in PTSD and depression symptoms, suggesting that none of these interventions as delivered are efficacious in reducing PTSD and depression symptoms, at least not over the course of a brief six-week intervention. This finding does not preclude the possibility that any one of these interventions has the potential to mitigate such symptoms using more protracted treatment, or with these interventions applied using different techniques.

In summary, the present results suggest that Tennant Biomodulator treatment, traditional Chinese acupuncture, and TENS may decrease symptoms of chronic pain. Because these interventions have few contraindications and carry very low risk—particularly in contrast to opioid medications—they can be considered stand-alone treatments or complements to other forms of chronic pain management. Importantly, the addition of these treatments to usual care may hypothetically decrease the need for opioid-based medications in patients with chronic pain. Although this relationship was not directly tested in the present study, evidence suggests that acupuncture [41, 42] and TENS [43, 44] may reduce opioid use in those with chronic pain. Future studies should test whether the Tennant Biomodulator, a new and under-studied device, can also attenuate the need for opioid medication. The Tennant Biomodulator may have value as a nonpharmacological pain and associated symptom management solution because although the present findings suggest it is as equally efficacious as acupuncture or TENS, it addresses several of the limitations of basic TENS units, and unlike acupuncture, it does not require access to health care providers. Thus, patients may be able to self-manage pain at relatively low cost.

In interpreting these results, several study limitations should be considered. 1) The goal of the present study was to compare the Tennant Biomodulator to established, nonpharmacological pain management interventions within a pragmatic study design that reflects the nonpharmacological options that are normally available to patients with chronic pain. Therefore, the results should be interpreted as comparative efficacy results, rather than stand-alone efficacy results, which would require testing with a control or placebo arm. Although the goal of the present study was to compare the Tennant Biomodulator to established, nonpharmacological pain management interventions and not to usual treatment or a placebo, a control and/or a placebo group would nevertheless have strengthened the study design. 2) This study tested the Tennant Biomodulator on its most basic setting for in-home use; thus, the results do not necessarily reflect the full potential of this device. Similarly, singular acupuncture and TENS protocols were utilized. It is possible that different acupuncture approaches and TENS devices used at other frequencies may produce different comparative results. 3) This study had a high attrition rate, with 27% of the participants missing ≥4 of 6 clinical sessions, although this attrition should be interpreted in context; approximately half the study participants were active duty service members who have tight, highly regimented schedules and are often deployed at very short notice. It is likely that many of these participants were lost due to the demands of the military service, though the reasons participants were lost were not assessed. It should be noted that among clinical intervention trials in military populations, dropout rates of 30–40% are not uncommon (e.g., [24, 45]). 4) The treatment arms had differing attrition rates. Although no adverse events were reported throughout the course of the study, the Biomodulator and acupuncture treatment arms had higher attrition rates than the TENS arm. It is possible that participants found the TENS treatment to be less burdensome or uncomfortable, though in terms of their application, TENS and Biomodulator units are very similar, and Biomodulator treatment does not cause physical discomfort. Although intent-to-treat analyses were performed, it is not possible to assess how the drop-out rate affected the study outcomes. 5) Home-use compliance of the Biomodulator and TENS units was not assessed, meaning dose effects could not be determined.

These limitations should be considered with the study’s strengths, which include high quality treatment interventions delivered by a medical doctor, a range of assessment measures exploring pain and its sequelae, and high ecological validity. The present findings suggest that the Tennant Biomodulator used on its most basic setting is as efficacious as traditional Chinese acupuncture and TENS stimulation at 2 Hz in reducing chronic pain and pain's functional sequelae. Given that the Tennant Biomodulator is accessible, scalable, and safe to use, has a lower reported side-effect profile than TENS, and does not require access to regular medical care, this device warrants further testing in large-scale, randomized controlled trials. Such trials should test this device across its full range of settings, ideally against control or placebo groups.

## Conclusions

The present study tested the efficacy of the Tennant Biomodulator—a novel pain management intervention using biofeedback-modulated electrical stimulation—in reducing chronic pain and pain's psychosocial sequelae in a sample of current and former military service members. The Tennant Biomodulator was compared to two commonly used, non-pharmacological pain treatments: traditional Chinese acupuncture, and acupuncture-like TENS with 2 Hz stimulation. The findings build on previous work suggesting that traditional Chinese acupuncture and TENS can reduce pain and its functional sequelae without risks associated with pharmacological pain management. The Tennant Biomodulator, used here on its most basic setting, performed as well as these interventions. Based on the present findings, large, randomized controlled trials on the Tennant Biomodulator are indicated. Future work should also test this device using its full range of settings for pain-related psychological health.

## Data Availability

The datasets used and/or analyzed during the current study are available from the author W. Zhang upon reasonable request.

## Abbreviations

CES-D: The Center for Epidemiological Studies - Depression Scale
MVAS: Million Visual Analog Scale
PCL-M: PTSD Checklist – Military
PTSD: Post-traumatic stress disorder
TENS: Transcutaneous electrical nerve stimulation
